# Transcriptomic and metabolomic analyses of root responses in *Indigofera stachyodes* seedlings under drought stress: a medicinal plant native to karst mountainous regions

**DOI:** 10.3389/fpls.2025.1607789

**Published:** 2025-07-01

**Authors:** Qingqing Ye, Na Zhang, Xin Tan, Li Yang, Ning Ding, Wei Zhou, Zhikun Wu

**Affiliations:** ^1^ Department of Pharmacy, Guizhou University of Traditional Chinese Medicine, Guiyang, China; ^2^ Department of Pharmacy, Tongren Hospital of Traditional Chinese Medicine, Tongren, China; ^3^ Germplasm Bank of Wild Species & Yunnan Key Laboratory of Crop Wild Relatives Omics, Kunming Institute of Botany, Chinese Academy of Sciences, Kunming, China

**Keywords:** Indigofera stachyodes, drought stress, transcriptome, metabolome, karst mountainous regions

## Abstract

**Introduction:**

*Indigofera stachyodes* Lindl. is a perennial shrub belonging to the Fabaceae family that has been traditionally utilized as a medicinal plant by ethnic minority groups in Guizhou Province, China. This species exhibits significant ethnopharmacological value in local traditional medicine systems. The plant predominantly inhabits karst mountainous regions characterized by frequent drought stress, which represents a typical harsh habitat for plant growth. Notably, drought conditions particularly impair the establishment and development of *I. stachyodes* seedlings. However, the molecular mechanisms underlying its drought tolerance and adaptive responses remain largely unexplored, warranting further investigation at the molecular level.

**Methods:**

We conducted pot-based water control experiments to subject *I. stachyodes* seedlings to drought stress treatments (CK, T0, T2). Root tissues from each treatment group were analyzed using transcriptomics (RNA-seq) and metabolomics (LC-MS/GC-MS) approaches to identify differentially expressed genes (DEGs) and differentially expressed metabolites (DEMs). Through integrated analysis of DEGs and DEMs, we performed KEGG pathway enrichment and constructed co-expression networks to elucidate the molecular mechanisms underlying drought stress responses in the roots of *I. stachyodes* seedlings.

**Results:**

A total of 11,509 DEGs were detected in the transcriptome. Among them, the CK vs T0 group shared 7,191 DEGs, the CK vs T2 group shared 1,264 DEGs, and the T2 vs T0 group shared 3,054 DEGs. In the metabolome, a total of 622 metabolites were detected. Among them, the CK vs T0 group shared 187 DEMs, the CK vs T2 group shared 127 DEMs, and the T2 vs T0 group shared 86 DEMs. The transcriptome-metabolome analysis revealed that the roots of *I. stachyodes* seedlings regulate metabolic balance through the phenylpropanoid biosynthesis pathway and the flavonoid biosynthesis pathway when subjected to varying degrees of drought stress. Metabolites such as p-coumaric acid, sinapine malate, eugenol, coumestrol, medicarpin, prunin, isosakuranetin, vitexin, gallocatechin, catechin, garbunzol and dihydromyricetin, along with genes including *PAL*, *C4H*, *COMT*, *4CL*, *CHS*, *DFR*, *HIDH*, *I2’H*, *IF7GT*, *IF7MAT*, *IFR*, *VR*, *PTS* and *IFS* are potential key substances that enable the roots of *I. stachyodes* seedlings to resist drought stress.

**Discussion:**

These results elucidate that the roots of *I. stachyodes* seedlings can resist drought stress and adapt to drought environments by regulating the expression of genes and the synthesis of metabolites in the flavonoid and phenylpropanoid metabolic pathways, providing a foundation to facilitate the domestication of wild *I. stachyodes.*

## 1 Introduction

*Indigofera stachyodes* Lindl., a species of the genus *Indigofera* (family Fabaceae), is primarily distributed in the karst mountainous regions of Guizhou, Yunnan, and Guangxi in China, where the plant has been traditionally used as a medicinal resource by ethnic minority communities. Its dried root is a renowned medicinal material known as “Blood Ginseng” (Xue Renshen), one of the top ten Miao ethnic medicines in Guizhou Province. It possesses the effects of tonifying deficiency, promoting blood circulation, securing collapse, draining dampness, nourishing yin, and resolving phlegm. It is primarily used to treat conditions such as chronic diarrhea due to physical weakness, metrorrhagia and metrostaxis, rheumatic arthralgia, cold and fever, cough, non-healing ulcers, traumatic injuries, and infantile malnutrition (Chen et al., 2014). In addition, Qijiao Shengbai capsule, which is produced using *I. stachyodes* as the main ingredient, has shown clinical efficacy in treating leukemia (Zhao et al., 2020). Currently, the medicinal material derived from *I. stachyoides* is predominantly obtained from wild sources. The environment in which these wild populations exist has a direct impact on their survival and expansion. Furthermore, the ability of seedlings to adapt to the ecological conditions of their natural habitat significantly influences both their survival rates and the overall development of the plant population.

Water is essential for plant growth, development, and physiological metabolism. However, approximately one-third of the world’s land area and half of China’s territory are in arid or semi-arid conditions (Wen et al., 2020). Plants undergo phenotypic, biochemical, and molecular-level changes when facing drought stress. In severe cases, this can lead to the cessation of photosynthesis, metabolic disorders, and even plant death (Jaleel et al, 2008). Therefore, research on plant adaptation to water environments is of great significance. During medicinal plant growth, root systems experience drought stress when water supply-demand balance is disrupted. The roots are the most sensitive and the first organ to respond to such conditions (Maurel and Nacry, 2020). Recent studies on medicinal plant responses to drought stress have primarily investigated root systems. Representative examples include: PEG-6000-induced stress effects on tanshinone accumulation in *Salvia miltiorrhiza* hairy roots (Sheng and Chen, 2013); effect of drought stress on root morphology and physiological characteristics of *Malus micromalus* cv. ‘Ruby’ (Zheng et al, 2020); and transcriptome sequencing-based analysis of gene expression regulation in *Glycyrrhiza uralensis* roots under moderate drought stress (Zhang et al., 2015).

With the rapid development of next-generation DNA sequencing technologies and systems biology, multi-omics approaches have become indispensable research methodologies. These techniques provide systematic and cellular-level insights into the dynamic changes occurring during plant growth and development. Among them, transcriptomics and metabolomics stand out as relatively mature, in-depth, and technically accessible platforms (Wu et al., 2023). Transcriptomics enables high-throughput acquisition of RNA-level gene expression information, revealing intrinsic connections between gene expression patterns and biological phenomena. Metabolomics serves as the critical bridge between genotype and phenotype, providing systematic analysis of all low-molecular-weight metabolites in tissues, organs, or cells to identify significantly differential metabolites with biological importance. Integrated transcriptomic-metabolomic analysis allows comprehensive investigation of plant physiological changes from both causal (transcriptional regulation) and consequential (metabolic output) perspectives. This approach facilitates the identification of key pathways associated with metabolic alterations and subsequent construction of core regulatory networks, thereby elucidating their underlying mechanisms (Wang et al., 2015; Duan et al., 2016; Xue et al., 2022). Currently, integrated transcriptomic-metabolomic analysis has been widely applied in plant abiotic stress research. Representative studies include the mechanistic elucidation of drought resistance in *Gossypium hirsutum* through integrated transcriptomic-metabolomic profiling (Zhang, 2018), an investigation into the drought resistance of tartary buckwheat based on metabolomics and transcriptomics (Ouyang, 2020), and the phosphorus-mediated modulation of ionomic-metabolic networks under drought stress in *Triticum aestivum* seedlings (Li et al., 2022).

Therefore, to examine the effects of drought stress on the biosynthesis of root secondary metabolites in *I. stachyodes* seedlings, this study performed integrated transcriptomic and metabolomic analyses on root samples subjected to drought stress. The objective is to elucidate the response mechanisms from the secondary metabolites in *I. stachyodes* roots under drought conditions, thereby providing foundational data to facilitate the domestication of wild *I. stachyodes.*

## 2 Materials and methods

### 2.1 Plant material

The seeds utilized in this study were collected from the Yue-liang River in Liuzhi, Guizhou Province, China. They were then germinated under controlled conditions within growth chambers and subsequently cultivated in a greenhouse with a photoperiod of 16 hours light and 8 hours dark. After reaching stable growth stage, uniformly developed seedlings were selected for pot-based drought stress experiments. Three gradients of field soil water holding capacity were established: 0–5% relative field capacity (extreme drought, T0), 40–45% relative field capacity (moderate drought, T2), and 80–85% relative field capacity (CK, control group). Soil moisture content was measured using the weighing method. The water stress treatment lasted for 30 days. After the experiment, root samples from seedlings in the T0, T2, and CK groups were randomly collected, with three replicates per treatment. The samples were immediately frozen in liquid nitrogen, stored in cryotubes, and preserved at −80°C for subsequent RNA and metabolite extraction.

### 2.2 Transcriptome sequencing and data analysis

RNA extraction was performed using the tengen biotech RNA extraction kit (DP441) according to the manufacturer’s protocol. The cDNA library was constructed and subjected to quality control using illumina’s NEBNext^®^ Ultra™ RNA library prep kit. After quality inspection, sequencing was carried out on the illumina platform. High-quality clean reads were obtained from the raw reads using the fastp tool to remove low-quality bases and N bases. Trinity and corset software were used to assemble and cluster transcripts to remove redundancy. Subsequently, the corresponding amino acid sequences were predicted from the processed transcripts.

DIAMOND software was then utilized to align the redundant transcript sequences against seven databases: kyoto encyclopedia of genes and genomes (KEGG), non-redundant protein sequence database (NR), curated protein sequence database (Swiss-Prot), gene ontology (GO), clusters of orthologous groups (COG/KOG), translated EMBL nucleotide sequence database (TrEMBL), and protein family database (PFAM) to generate functional annotations for the transcripts. The expression levels of transcripts were calculated using RNA-Seq by expectation-maximization (RSEM) software and quantified based on fragments per kilobase of transcript per million mapped reads (FPKM) values. Differential expression analysis between groups was performed using DESeq2 (Love et al., 2014; Huson and Buchfink, 2015; Varet et al., 2016). Differentially expressed genes (DEGs) were identified based on the thresholds of |log2fold change (FC)| ≥ 1 and FDR < 0.05. Subsequently, GO and KEGG enrichment analyses were conducted to determine the main biological functions and pathways associated with the DEGs.

### 2.3 Metabolomic profiling and data analysis

Samples from each treatment group were ground into powder and extracted with methanol. The extracts were vortexed, centrifuged, and filtered to obtain the supernatant, which was stored for further analysis. The samples were analyzed using an ultra-performance liquid chromatography-tandem mass spectrometry (UPLC-MS/MS) system equipped with a triple quadrupole detector. The UPLC conditions included optimized parameters for column type, mobile phase composition, elution gradient, flow rate, column temperature, and injection volume. The MS/MS analysis was performed with optimized ion source settings, including temperature, voltage, and gas flow rates. Qualitative and quantitative analyses of metabolites were conducted using the metabolomics workbench database (MWDB) and multi-reaction monitoring following standard protocols. To monitor technical variability during metabolomic profiling, a pooled quality control (QC) sample was prepared by combining equal aliquots (10 μL) from all experimental samples. The QC sample was injected repeatedly (n=5) at the beginning of the analytical sequence to equilibrate the system, followed by periodic injections after every 10 experimental samples throughout the LC-MS/MS run. This QC strategy enabled comprehensive monitoring of instrument stability, evaluation of data reproducibility, correction of batch effects, and filtration of technical noise.

Principal component analysis (PCA) and orthogonal partial least squares discriminant analysis (OPLS-DA) were performed on the metabolite data using the statistical functions in R software. These analyses aimed to identify differences in metabolites among samples, assess variation within groups, filter out noise, highlight inter-group differences, and facilitate the screening of differentially expressed metabolites (DEMs). The relative content of metabolites was normalized using the unit variance scaling method (UV). Hierarchical cluster analysis (HCA) was then performed, and heatmaps were generated using the pheatmap software package.

DEMs were screened by combining FC values and variable importance in projection (VIP) values from the OPLS-DA model, with thresholds set at |log2FC| ≥ 1, *P* < 0.05, and VIP > 1. Subsequently, KEGG enrichment analysis was performed on the identified DEMs to explore their associated biological pathways.

### 2.4 Integrated analysis of transcriptome and metabolome data

The results of metabolomic DEMs and transcriptomic DEGs were subjected to co-expression analysis and mapped onto KEGG pathways to identify biological pathways simultaneously affected at both transcriptional and metabolic levels under drought stress in *I. stachyodes* seedlings. This integrated approach comprehensively reveals the intrinsic connections between molecular regulation and physiological metabolic changes in the root system under drought conditions.

## 3 Results

### 3.1 Transcriptomic sequencing and analysis

The sequencing generated a total of 416,171,080 clean reads, corresponding to 62.42 Gb of high-quality clean bases. All libraries exhibited Q30 scores above 92.91%, with GC content ranging from 42.06% to 44.30%. *De novo* assembly using Trinity software produced 176,876,362 mapped reads, with mapping rates exceeding 80.66% (**Supplementary Table S1**). These results demonstrate high sequencing data saturation and excellent assembly performance. Pairwise correlation analysis revealed high reproducibility among replicates, with Pearson correlation coefficients ranging from 0.73 to 0.99 (**Supplementary Figure S1**). These results demonstrate strong inter-sample consistency, confirming the technical reliability of our transcriptomic datasets for subsequent analyses.

### 3.2 Analysis of DEGs between different treatments

**Figure 1:**
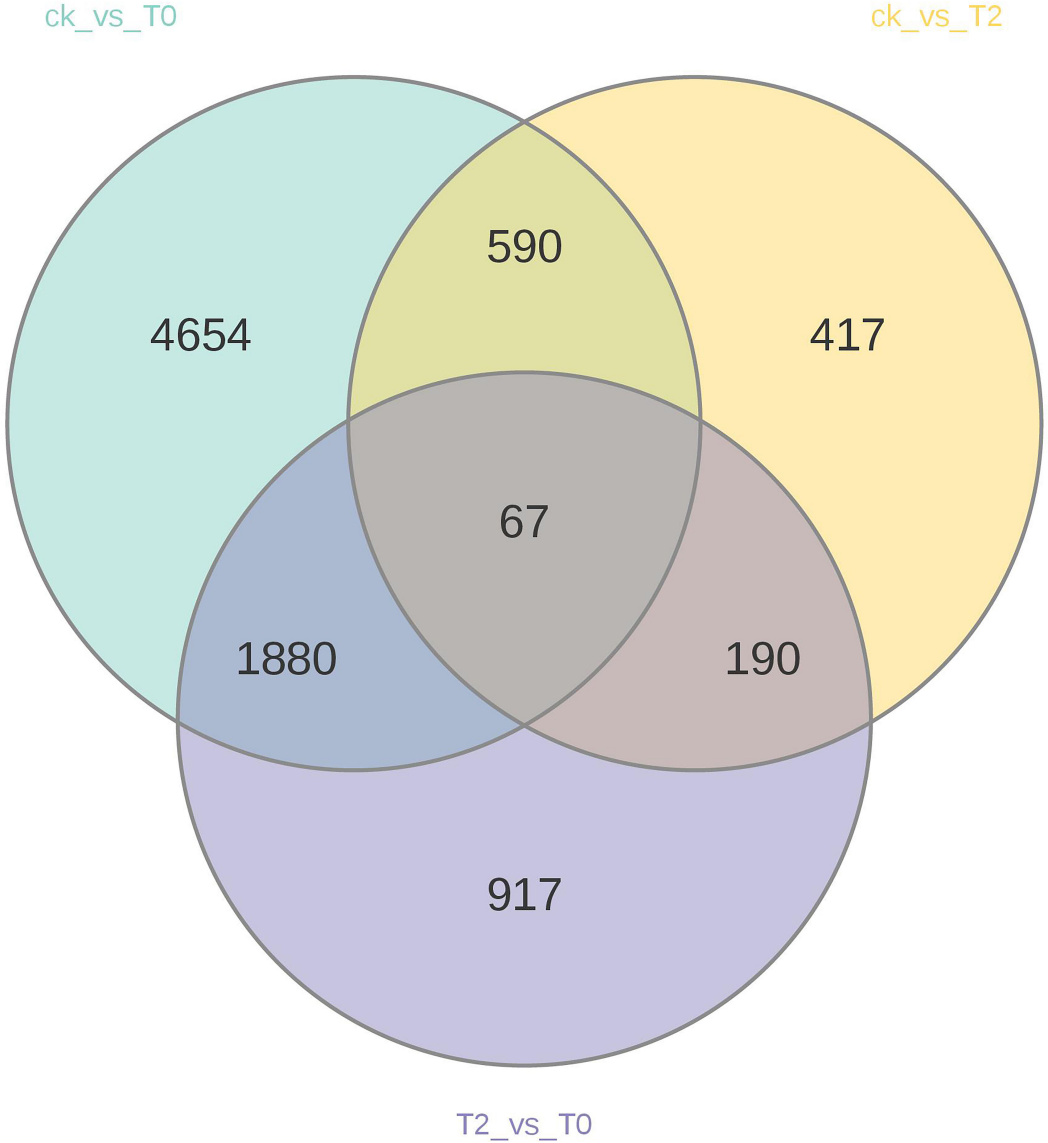
Venn diagram of differentially expressed genes (DEGs) analysis in each group.

The results show that a total of 11,509 DEGs were identified, including 5,835 up-regulated and 5,674 down-regulated genes. Specifically, the CK vs T0 group contained 7,191 DEGs (3,769 up-regulated and 3,422 down-regulated), the CK vs T2 group had 1,264 DEGs (498 up-regulated and 766 down-regulated), and the T2 vs T0 group exhibited 3,054 DEGs (1,568 up-regulated and 1,486 down-regulated). (**Supplementary Table S2**). Venn diagram analysis (**Figure 1**) revealed 67 common DEGs shared among all three treatment groups, while each comparison group exhibited unique DEG profiles: 4,654 (CK vs T0), 417 (CK vs T2), and 917 (T2 vs T0) group-specific DEGs. These results demonstrate distinct transcriptional reprogramming in *I. stachyodes* seedling roots under progressive drought stress intensities, with the number of responsive DEGs increasing significantly as drought severity escalates.

**Figure 2:**
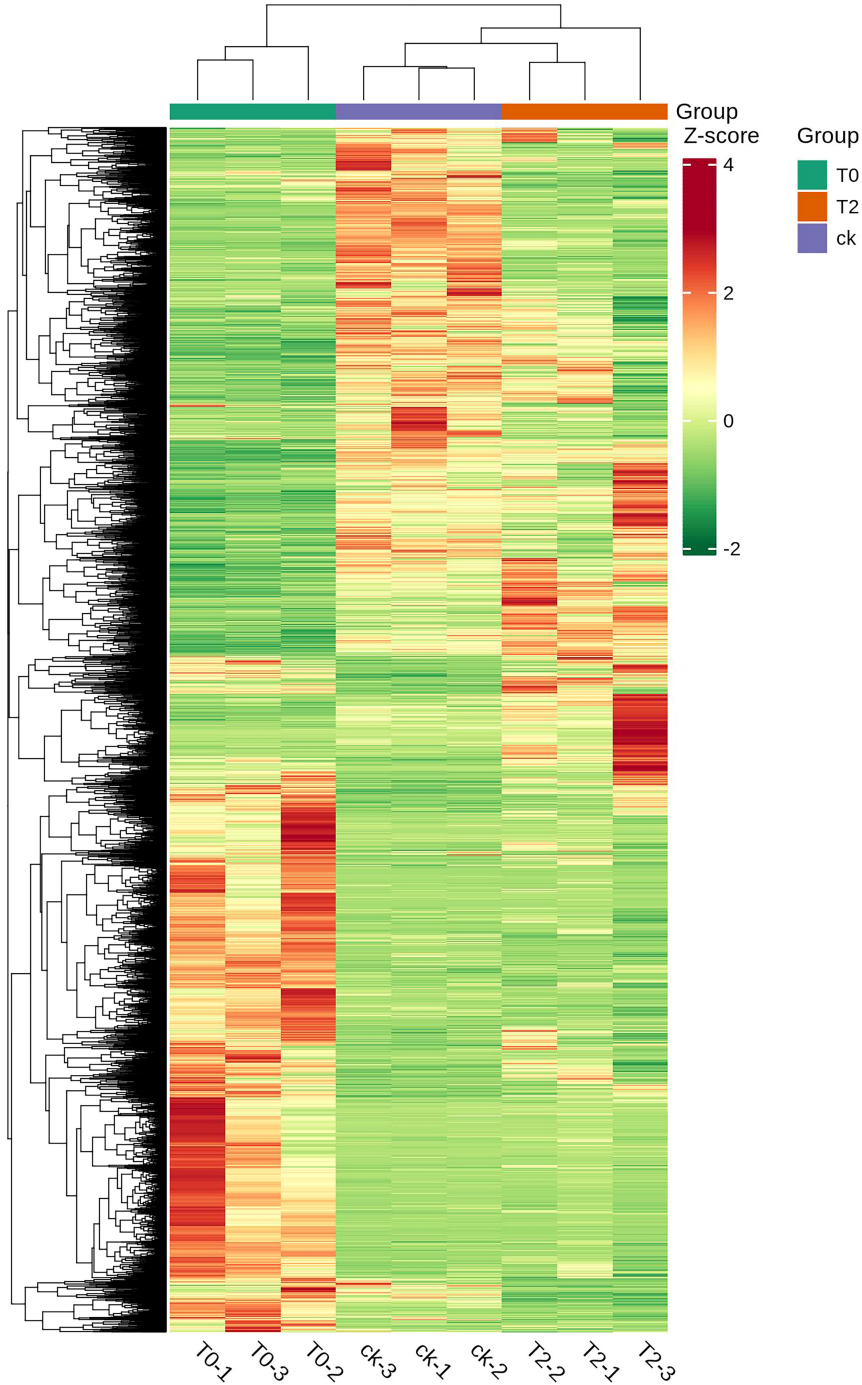
Hierarchical cluster analysis (HCA) of DEGs expression in each group.

HCA (**Figure 2**) revealed distinct expression patterns of DEGs across water stress treatments. Based on the expression levels of DEGs in each group under different degrees of drought treatment, the three treatment groups can be divided into two categories: T2 and CK are clustered into one category, while T0 forms a separate category. It is evident that the DEGs in the roots of *I. stachyodes* seedlings exhibited significant differences under varying degrees of drought stress.

### 3.3 GO functional enrichment analysis of DEGs

GO functional enrichment analysis revealed that in the CK vs T0 comparison group, a total of 4,965 DEGs were enriched, with 431 DEGs showing significant enrichment (P<0.05). These included 231 DEGs involved in biological processes, 172 in molecular functions, and 28 in cellular components. Among the top 20 most significantly enriched terms (lowest P-values), 9 were associated with biological processes-primarily related to phenylpropanoid and flavonoid biosynthesis and metabolic pathways; 5 were linked to cellular components-mainly ribosomal subunits; and 6 were connected to molecular functions predominantly enzyme activity-related terms.

The GO enrichment analysis identified 2,422 DEGs in the CK vs T2 comparison group, with 252 DEGs showing significant enrichment (P<0.05). These included 162 DEGs involved in biological processes, 11 in cellular components, and 79 in molecular functions. Among the top 20 most significantly enriched terms (lowest P-values), analysis revealed: 10 terms associated with biological processes-primarily cellular response to hypoxia; 1 term linked to cellular components-specifically the anchored component of membrane; and 9 terms connected to molecular functions-mainly inhibition of peptidase and endopeptidase activities.

GO functional enrichment analysis of the T2 vs T0 comparison identified 3,933 DEGs, with 326 showing significant enrichment (P<0.05). These included 186 DEGs associated with biological processes, 120 with molecular functions, and 20 with cellular components. Among the top 20 most significantly enriched terms (lowest P-values), analysis revealed: 8 biological processes-primarily cellular response to oxygen levels; 6 cellular components-mainly ribosomal subunits; and 6 molecular functions-predominantly translation elongation factor activity and water channel activity.

### 3.4 KEGG pathway enrichment analysis of DEGs

**Figure 3:**
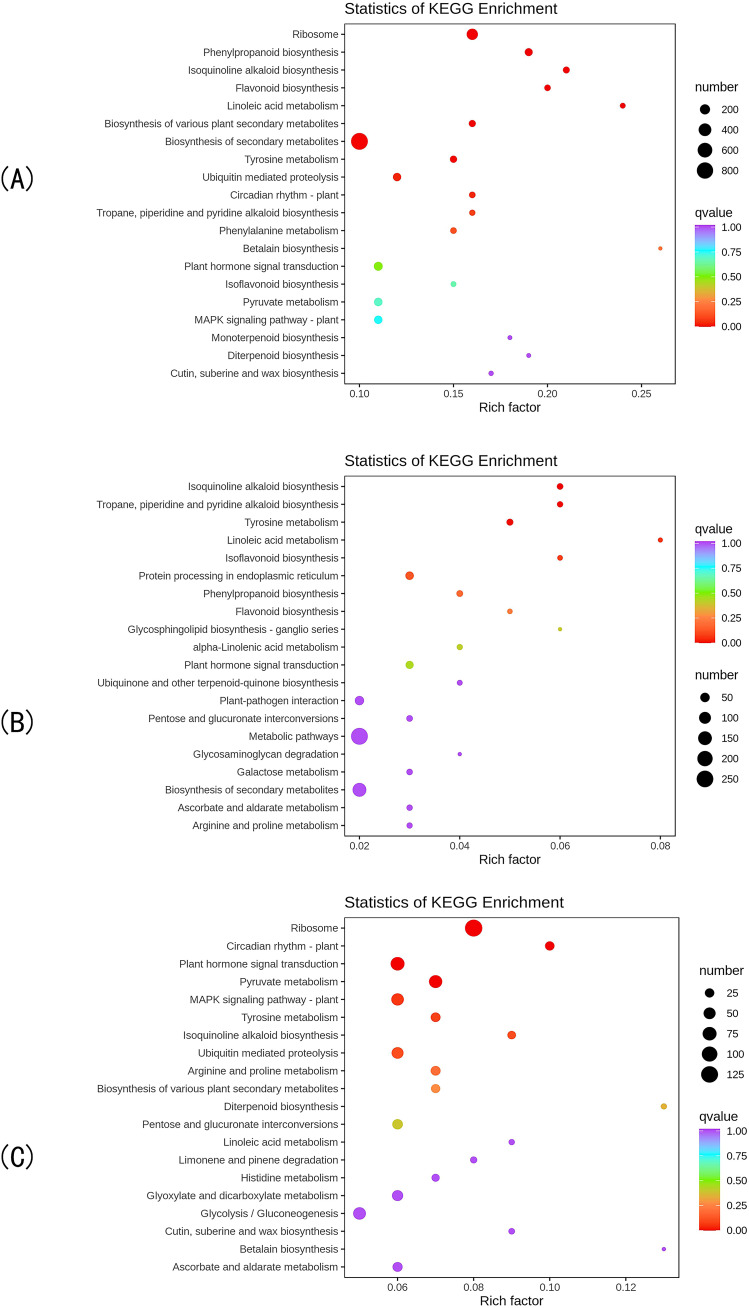
The top 20 pathways with the smallest KEGG enrichment P-values of DEGs in the CK vs T0 group **(A)**, CK vs T2 group **(B)**, and T2 vs T0 group **(C)**.

KEGG enrichment analysis revealed that the DEGs in the CK vs T0 comparison group were mapped to 140 metabolic pathways, with 27 pathways showing significant enrichment (P<0.05). The top 20 most significantly enriched pathways (**Figure 3A**) included: ribosome biogenesis in eukaryotes, phenylpropanoid biosynthesis, isoquinoline alkaloid biosynthesis, flavonoid biosynthesis, phenylalanine metabolism, and isoflavonoid biosynthesis. These results demonstrate that drought stress induces substantial metabolic reprogramming in *I. stachyodes* seedling roots, particularly affecting flavonoid metabolism, phenylpropanoid pathways, and phenylalanine-derived secondary metabolite biosynthesis.

KEGG pathway analysis identified 123 metabolic pathways enriched with DEGs in the CK vs T2 comparison, among which 20 pathways showed significant enrichment (P<0.05). The top 20 most significantly enriched pathways (**Figure 3B**) primarily included: isoflavonoid biosynthesis, isoquinoline alkaloid biosynthesis, flavonoid biosynthesis, phenylpropanoid biosynthesis, and linoleic acid metabolism. These findings indicate that moderate drought stress triggers substantial metabolic reprogramming in *I. stachyodes* seedling roots, particularly affecting the biosynthesis pathways of flavonoids, isoflavonoids, alkaloids, and fatty acid derivatives.

KEGG pathway enrichment analysis revealed 136 metabolic pathways associated with DEGs in the T2 vs T0 comparison group, with 22 pathways demonstrating significant enrichment (P<0.05). The top 20 most significantly enriched pathways (**Figure 3C**) included: ribosome, plant hormone signal transduction, pyruvate metabolism, isoquinoline alkaloid biosynthesis, and ubiquitin mediated proteolysis. These results demonstrate that progressive drought stress induces substantial metabolic reprogramming in *I. stachyodes* seedling roots, particularly affecting: translational machinery (ribosomal pathways), stress hormone signaling networks, central carbon metabolism, and specialized metabolite biosynthesis. The coordinated regulation of these pathways suggests a hierarchical adaptive strategy in response to increasing drought severity.

### 3.5 Metabolomics PCA and OPLS-DA

**Figure 4:**
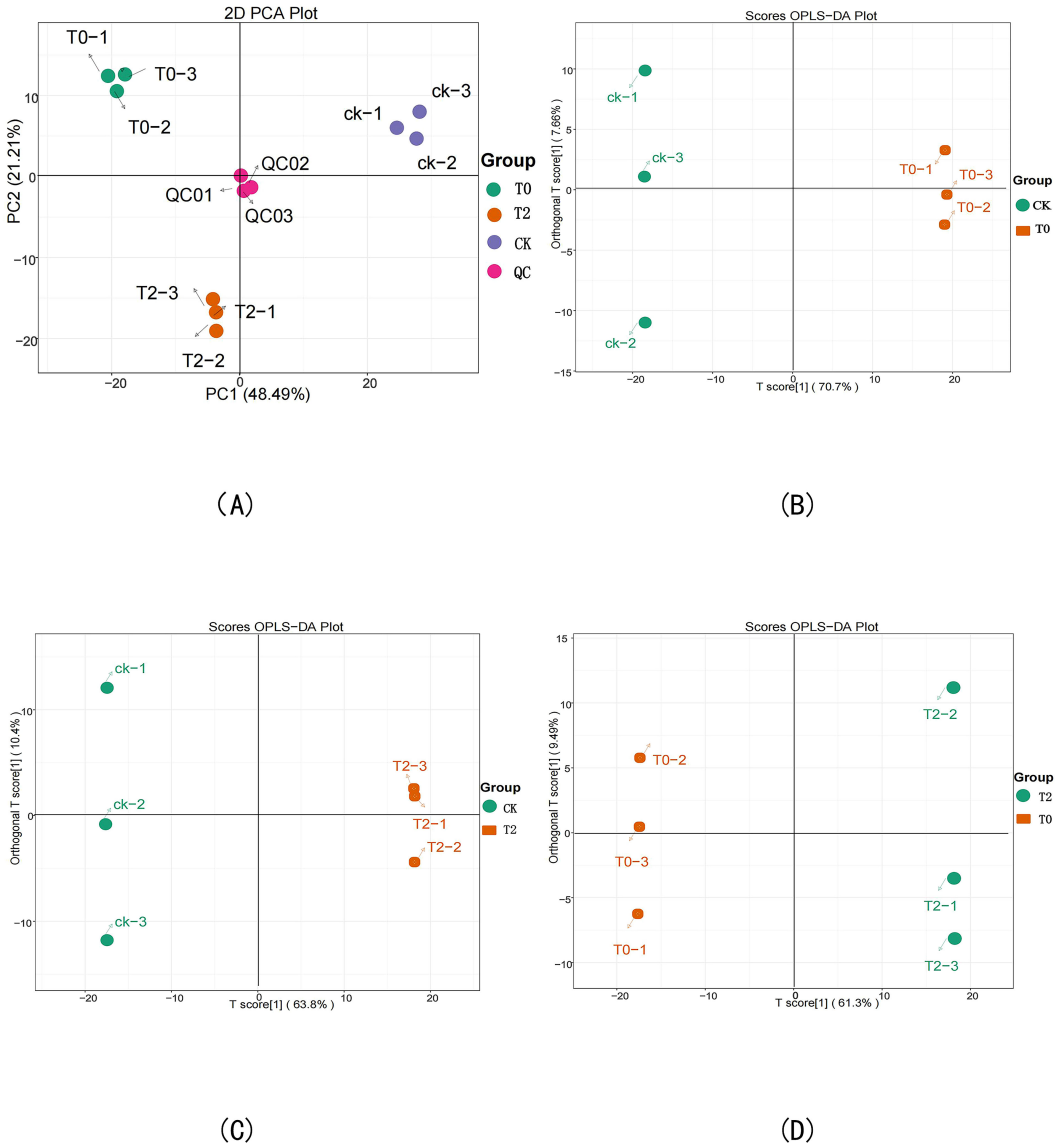
Principal component analysis (PCA) of sample metabolome test results **(A)**, OPLS-DA score plots of CK vs T0 **(B)**, CK vs T2 **(C)**, and T2 vs T0 **(D)**.

PCA results derived from different drought stress treatments and QC sample revealed minimal variation among biological replicates within each treatment group, while showing distinct separation trends between different treatment groups. These findings demonstrate significant metabolic differences among drought stress treatments. Pairwise comparative analysis further indicated clear separation along both PC1 and PC2 axes between treatment groups, confirming that drought stress significantly alters the metabolic profile of *I. stachyodes* (**Figure 4A**). After filtering out noise unrelated to classification information using the OPLS-DA model, the sample groups exhibited clear separation trends in the OPLS-DA score plot (**Figures 4B, C, D**). In plot B, all samples of the CK group were distributed on the left side, while those of the T0 group were entirely located on the right. In plot C, all CK group samples were clustered on the left, whereas the T2 group samples were entirely positioned on the right. Similarly, in plot D, all T0 group samples were distributed on the left, and the T2 group samples were entirely located on the right. These results indicate significant differences in metabolites among the sample groups and demonstrate the reliability of the OPLS-DA model, which can be effectively used for screening DEMs.

### 3.6 Metabolite detection results

**Figure 5:**
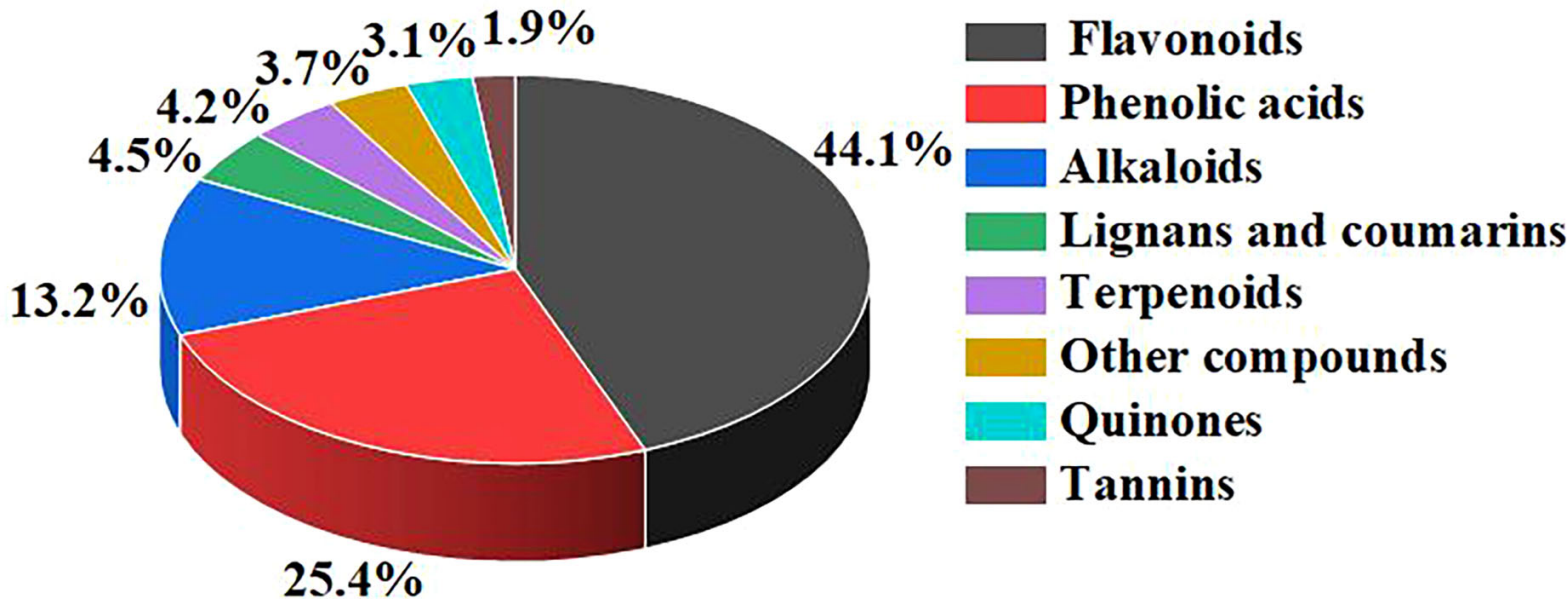
Metabolite classification map of sample metabolome test results.

In this study, a total of 622 metabolites were detected using the UPLC-MS/MS platform and the database established by Wuhan metware biotechnology Co., Ltd. (**Figure 5**). These metabolites were classified into 8 categories, with their respective proportions as follows: flavonoids (44.05%, 274/622), phenolic acids (25.4%, 158/622), alkaloids (13.18%, 82/622), lignans and coumarins (4.5%, 28/622), terpenoids (4.18%, 26/622), other compounds (3.7%, 23/622), quinones (3.05%, 19/622), and tannins (1.93%, 12/622).

### 3.7 Analysis of DEMs between treatments

Using the screening criteria of VIP ≥ 1 and P < 0.05 for DEMs, pairwise comparative analyses between groups were conducted, yielding the following results (**Table 1**): a total of 127 DEMs were detected in the CK vs T2 group, including 37 up-regulated and 90 down-regulated metabolites. Among the up-regulated DEMs, there were 13 flavonoids, 16 phenolic acids, 4 alkaloids, 2 lignans and coumarins, and 2 quinones. The down-regulated DEMs mainly included 40 flavonoids, 24 phenolic acids, 6 alkaloids, and 9 terpenoids. A total of 187 DEMs were detected in the CK vs T0 group, including 65 up-regulated and 122 down-regulated metabolites. The up-regulated DEMs mainly included 16 flavonoids, 25 phenolic acids, 12 alkaloids, 6 lignans, and 6 coumarins. The down-regulated DEMs mainly consisted of 66 flavonoids, 31 phenolic acids, and 7 alkaloids. A total of 86 DEMs were detected in the T2 vs T0 group, including 39 up-regulated and 47 down-regulated metabolites. Among the up-regulated DEMs, there were mainly 9 flavonoids, 10 phenolic acids, and 9 alkaloids. The down-regulated DEMs mainly consisted of 32 flavonoids, 7 phenolic acids, and 3 alkaloids.

**Table 1:** Comparison of differentially expressed metabolites (DEMs) between groups.

Group Class	CK VS T2		CK VS T0		T2 VS T0	
	Up	Down	Up	Down	Up	Down
Flavonoids	13	40	16	66	9	32
Phenolic acids	16	24	25	31	10	7
Alkaloids	4	6	12	7	9	3
Terpenoids	0	9	4	6	6	1
Lignans and Coumarins	2	5	6	2	4	1
Quinones	2	2	2	4	1	1
Tannins	0	2	0	4	0	2
Others	0	2	0	2	0	0
Total	127		187		86	

Analysis of DEMs revealed that drought stress primarily affected the accumulation of flavonoids, phenolic acids, and alkaloids in the roots of *I. stachyodes* seedlings. The comparative results of DEMs between treatment groups showed that the number of down-regulated metabolites exceeded that of up-regulated metabolites under drought stress, with flavonoids and phenolic acids being the predominant affected classes. These findings demonstrate that drought stress predominantly influences the biosynthesis of flavonoids and phenolic acids in *I. stachyodes* seedlings.

### 3.8 KEGG metabolic pathway enrichment analysis

**Figure 6:**
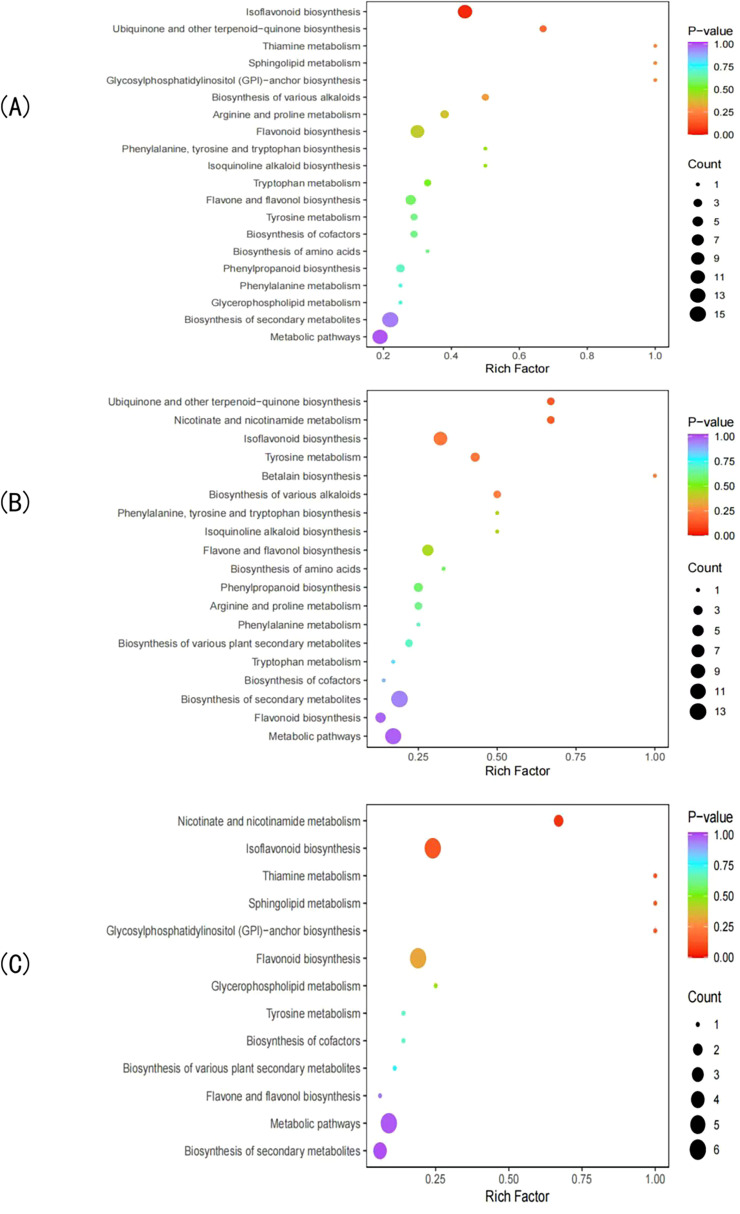
KEGG enrichment analysis of DEMs in the CK vs T0 **(A)**, CK vs T2 **(B)**, and T2 vs T0 **(C)** groups.

KEGG pathway enrichment analysis of significant DEMs between groups revealed that the CK vs T0 group showed predominant enrichment in several metabolic pathways (**Figure 6A**). These included isoflavonoid biosynthesis, biosynthesis of secondary metabolites, ubiquinone and other terpenoid-quinone biosynthesis, tyrosine metabolism, sphingolipid metabolism, flavonoid biosynthesis, and phenylpropanoid biosynthesis. These findings suggest that drought stress primarily affects these key metabolic pathways in *I. stachyodes* seedlings.

The KEGG pathway enrichment analysis of significant DEMs in the CK vs T2 group revealed predominant enrichment in several key metabolic pathways (**Figure 6B**). These included ubiquinones and other terpenoid-quinone biosynthesis, nicotinate and nicotinamide metabolism, isoflavonoid biosynthesis, tyrosine metabolism, betalain biosynthesis, and biosynthesis of various alkaloids. These results indicate that prolonged drought stress significantly impacts these specific metabolic networks in *I. stachyodes*, particularly affecting redox-related pathways (ubiquinone biosynthesis), secondary metabolite production (isflavonoids and alkaloids), and nitrogen metabolism (nicotinamide and tyrosine pathways).

The KEGG pathway enrichment analysis of significant DEMs in the T2 vs T0 group showed predominant enrichment in several crucial metabolic pathways (**Figure 6C**). These included nicotinate and nicotinamide metabolism, isoflavonoid biosynthesis, tyrosine metabolism, sphingolipid metabolism, and glycosylphosphatidylinositol-anchor biosynthesis. These findings suggest that the transition from moderate (T0) to prolonged (T2) drought stress in *I. stachyodes* primarily affects pathways related to: energy metabolism and redox homeostasis (nicotinate/nicotinamide), secondary metabolite production (isflavonoids), amino acid metabolism (tyrosine), and membrane structure and signaling (sphingolipids and GPI-anchored proteins).

### 3.9 Association analysis of transcriptome and metabolome

**Figure 7:**
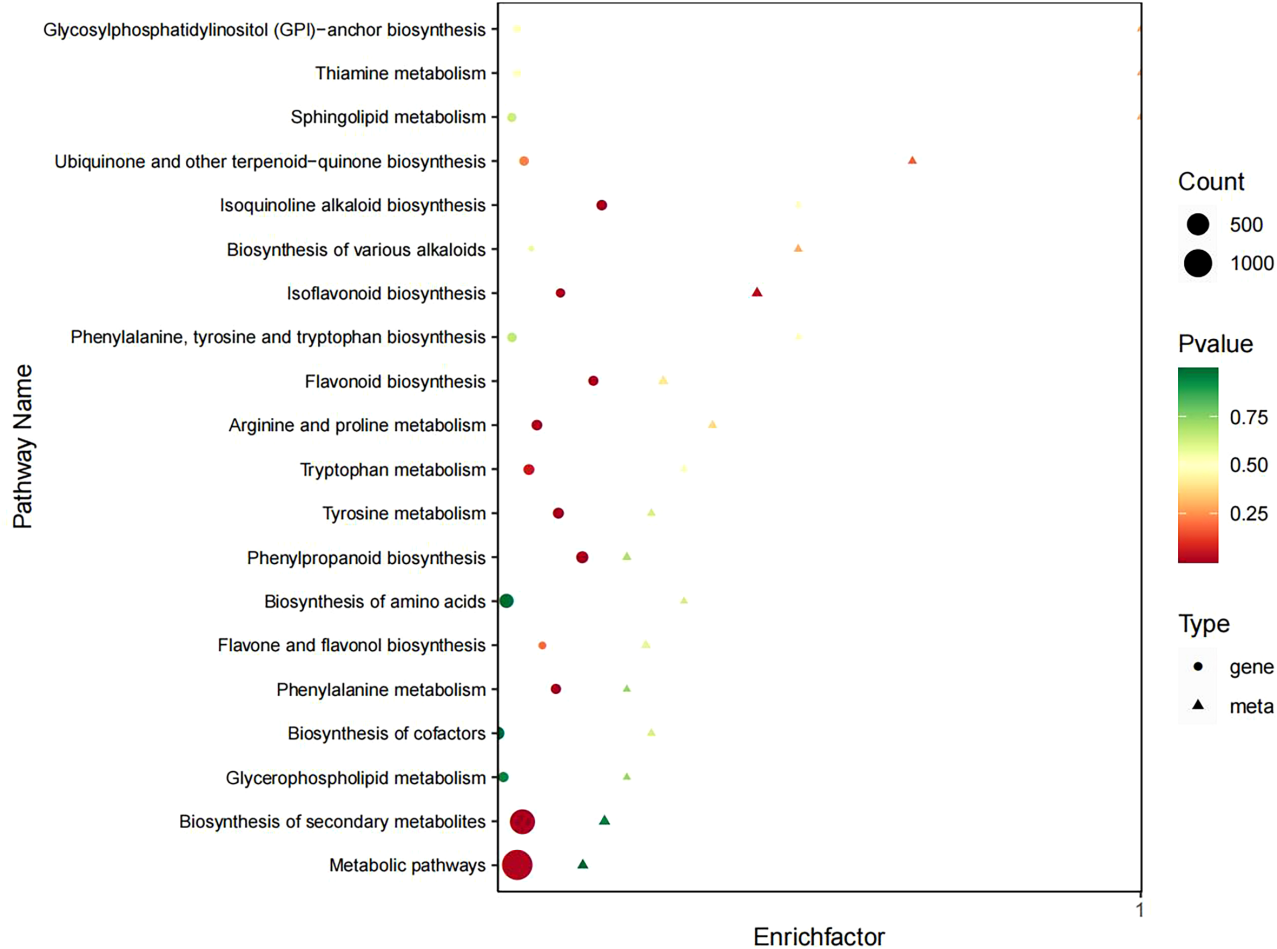
Pathway enrichment analysis of DEGs and DEMs.

Comparative analysis of DEGs and DEMs between drought-stressed groups (T0, T2) and the control group (CK) revealed that the CK vs T0 comparison exhibited the highest numbers of both DEGs and DEMs. Consequently, we performed integrated transcriptome-metabolome association analysis specifically for the CK vs T0 group. The results demonstrated that drought stress-induced DEGs in *I. stachyodes* seedling roots were predominantly associated with DEMs of flavonoids, phenolic acids, alkaloids, and lignins (**Supplementary Figure S2**). Both DEGs and DEMs were significantly enriched in key metabolic pathways including isoflavonoid biosynthesis, phenylpropanoid biosynthesis, and flavonoid biosynthesis (**Figure 7**), with the isoflavonoid and flavonoid biosynthesis pathways showing particularly prominent differential expression patterns. Based on these findings, we focused our subsequent analysis on the flavonoid biosynthesis-related metabolic pathways.

#### 3.9.1 Analysis of the phenylpropanoid biosynthesis metabolic pathway

**Figure 8:**
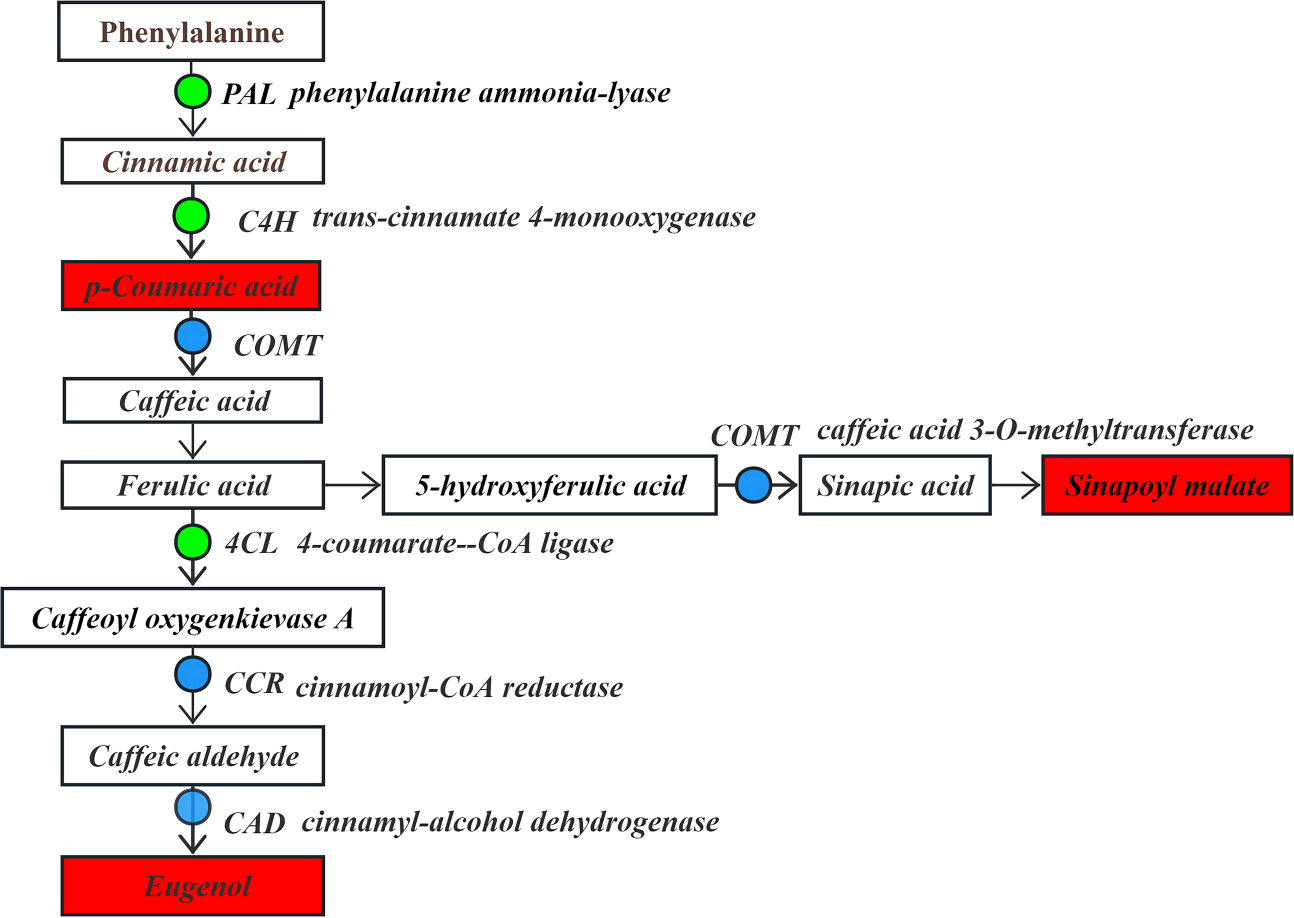
The expression levels of differential DEGs and DEMs in the phenylpropanoid biosynthesis pathway.

Note: The boxes represent DEMs, and the dots represent DEGs. Green indicates significantly downregulated, red indicates significantly upregulated, and blue indicates both up-regulation and down-regulation.

In the phenylpropanoid biosynthesis pathway (**Figure 8**), phenylalanine serves as the substrate. It is significantly down-regulated by phenylalanine ammonia-lyase (*PAL*) to generate the intermediate product cinnamic acid. Subsequently, the pathway is regulated by genes such as trans-cinnamate 4-monooxygenase (*C4H*), which is significantly down-regulated, caffeic acid 3-O-methyltransferase (*COMT*), cinnamoyl-CoA reductase (*CCR*) and cinnamyl-alcohol dehydrogenase (*CAD*), which are significantly up-regulated/down-regulated. These changes led to a significant up-regulation in the contents of p-coumaric acid, sinapine malate, and eugenol compounds. Moreover, the flavonoid biosynthesis pathway is significantly affected by the down-regulation of 4-coumarate-CoA ligase (*4CL*) using cinnamic acid as a substrate, making it an upstream regulator in the flavonoid biosynthesis pathway. The produced cinnamic acid serves as a key precursor involved in the flavonoid biosynthesis pathway.

#### 3.9.2 Analysis of the flavonoid biosynthetic metabolic pathway

**Figure 9:**
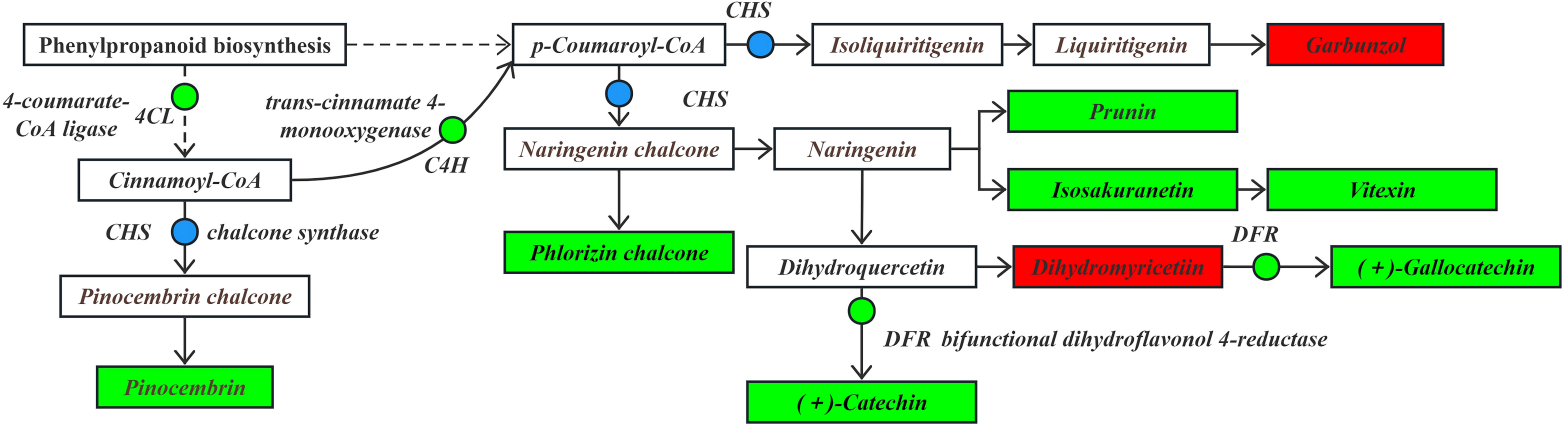
The expression levels of DEGs and DEMs in the flavonoid biosynthetic pathway.

Under drought stress, the flavonoid biosynthesis pathway (**Figure 9**) in the roots of *I. stachyodes* seedlings utilized cinnamoyl-CoA as the substrate. Chalcone synthase (*CHS*), which was significantly up-regulated/down-regulated, catalyzed the production of pinocembrin chalcone, indirectly leading to a significant down-regulation in the content of pinocembrin. Using the intermediate products cinnamoyl-CoA as substrates, the significant down-regulation by *C4H* eads to the production of p-coumaroyl-CoA. This compound is then significantly up-regulated/down-regulated by *CHS* to form isoliquiritigenin and naringenin chalcone, which are further converted into liquiritigenin and naringenin. Using liquiritigenin as an intermediate, the content of garbunzol is significantly up-regulated. In contrast, the contents of phloridzin chalcone, which are involved in the naringenin chalcone pathway, were significantly down-regulated.

In addition, in the apigenin synthesis pathway using naringenin as a substrate, the content of vitexin prunin, and isosakuranetin was significantly down-regulated. In the metabolic pathway where naringenin is used as a substrate to synthesize eriodictyol, and eriodictyol is further converted into dihydroquercetin, the content of dihydromyricetin was detected to be significantly up-regulated. However, through the significant down-regulation by dihydroflavonol 4-reductase (*DFR*), the content of gallocatechin was significantly down-regulated. Additionally, the content of catechin, which is directly produced from dihydroquercetin through the down-regulation of *DFR*, was also significantly down-regulated. Catechin and gallocatechin in flavonoids are among the key components for quality control of the *I. stachyodes* (Fu et al., 2013; Zhu et al., 2016). According to the results of this study, the content of catechin in the roots of *I. stachyodes* seedlings was significantly down-regulated under drought stress. Therefore, attention should be paid to soil moisture management during the transplanting of *I. stachyodes* seedlings.

Note: The boxes represent DEMs, and the dots represent DEGs. Green indicates significantly downregulated, red indicates significantly upregulated, and blue indicates both up-regulation and down-regulation.

#### 3.9.3 Analysis of the isoflavone biosynthetic metabolic pathway

**Figure 10:**
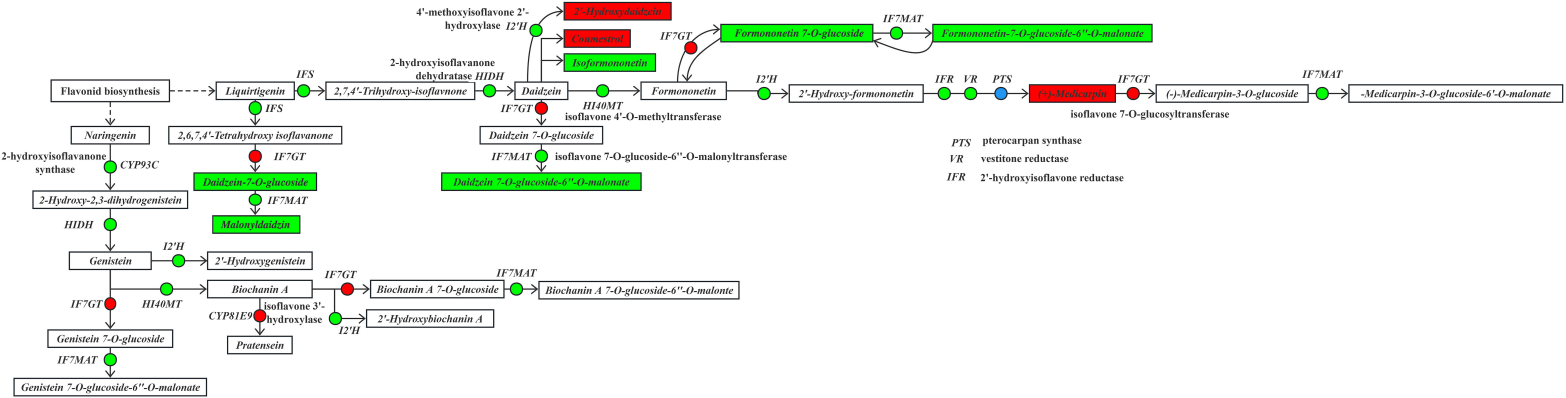
The expression levels of DEGs and DEMs in the isoflavone biosynthesis pathway.

Under drought stress, the isoflavone biosynthetic pathway (**Figure 10**) in the roots of *I. stachyoides* seedlings exhibited significant metabolic reprogramming when liquiritigenin from the flavonoid pathway served as the precursor. Key enzymatic perturbations included substantial down-regulation of both 2-hydroxyisoflavanone synthase (*IFS*) and 2-hydroxyisoflavone dehydratase (*HIDH*), resulting in the accumulation of 2,7,4’-trihydroxy-isoflavone and subsequent production of daidzein. These modifications triggered a marked increase in coumestrol content while significantly reducing isosakuranetin levels. When daidzein functioned as an intermediate substrate, the pathway showed pronounced suppression of formononetin-mediated isoflavone 2’-hydroxylase (*I2’H*) activity, leading to significantly increase 2’-hydroxydaidzein accumulation. Furthermore, daidzein is metabolized through the coordinated action of significantly up-regulated isoflavone 7-O-glucosyltransferase (*IF7GT*) and down-regulated isoflavone 7-O-glucoside-6″-O-malonyltransferase (*IF7MAT*), ultimately causing a substantial down-regulation in the production of daidzein 7-O-glucoside-6″-O-malonate. The daidzein-derived formononetin was subjected to significant up-regulation by *IF7GT*, resulting in a marked decrease in the production of formononetin 7-O-glucoside. Subsequently, the significantly down-regulated activity of *IF7MAT* led to a substantial reduction in the accumulation of formononetin 7-O-glucoside-6″-O-malonate. Using the intermediate formononetin as substrate, significant down-regulation of *I2’H* activity led to the production of 2’-hydroxymethy-formononetin. Subsequent coordinated enzymatic modifications including significant down-regulation of both 2’-hydroxyisoflavone reductase (*IFR*) and vestitone reductase (*VR*), along with significant up-regulation/down-regulation of pterocarpan synthase (*PTS*) collectively resulted in markedly increased accumulation of medicarpin.

In the isoflavone biosynthetic pathway, an alternative branch utilizing liquiritigenin as substrate proceeds through 6,7,4’-trihydroxyflavanone. Despite significant down-regulation of *IFS* activity, the production of 2,6,7,4’-tetrahydroxy-isoflavone remained unchanged. The daidzein generated through this pathway was subjected to significant up-regulation by *IF7GT*, resulting in markedly decreased accumulation of daidzein-7-O-glucoside. Subsequently, the significantly down-regulated activity of *IF7MAT* led to substantially reduced formation of malonyldaidzin.

Note: The boxes represent DEMs, and the dots represent DEGs. Green indicates significantly downregulated, red indicates significantly upregulated, and blue indicates both up-regulation and down-regulation.

#### 3.9.4 Analysis of flavone and flavonol biosynthesis metabolic pathways

No significant enrichment of DEGs was detected in this pathway, but the contents of isovitexin and vitexin, luteolin 7-O-β-D-glucosidic acid, astragalin and trifolin produced by apigenin, luteolin and kaempferol as substrates were significantly down-regulated (**Supplementary Figure S3**).

## 4 Discussions

Our previous studies have shown that under simulated drought stress on the growth of *I. stachyodes* seedlings, as the concentration of PEG-6000 increased, the shoot length, primary root length, number of fibrous roots, and fresh weight of *I. stachyodes* seedlings initially increased and then decreased (Yang et al., 2024a). Under extreme drought conditions, the malondialdehyde (MDA) content and free proline (Pro) content in *I. stachyodes* seedlings were the lowest, while catalase (CAT) activity was the strongest (Yang et al., 2024b). This indicates that mild drought can, to some extent, promote the growth and development of *I. stachyodes* seedlings, but intensified drought will inhibit their growth or even lead to death. In this study, we aim to explore the molecular-level response mechanisms of *I. stachyodes* seedlings to drought stress using transcriptomics and metabolomics.

Flavonoids are a major class of plant secondary metabolites with diverse biological functions, including antioxidant activity, free radical scavenging, anti-inflammatory effects, antiviral properties, and enhanced stress resistance (Nakabayashi et al., 2014). Structurally, flavonoids can be categorized into subgroups such as flavones, isoflavones, flavanones, and flavonols (Schijlen et al., 2004). Under drought stress, plants experience a significant accumulation of reactive oxygen species (ROS), leading to oxidative damage to cellular membranes, proteins, and nucleic acids. To counteract this, plants up-regulate flavonoid biosynthesis, which mitigates ROS-induced toxicity through their potent antioxidant capacity (Ahmed et al., 2021; Shomali et al., 2022). This protective mechanism enhances drought tolerance by reducing oxidative stress and maintaining cellular homeostasis (Foyer et al., 1994; Liu et al., 2024). For instance: in *citrus*, increased flavonol and anthocyanin biosynthesis has been shown to significantly boost antioxidant activity and drought resilience (Zandalinas et al., 2017). *Scutellaria baicalensis* exhibits elevated flavonoid accumulation and enhanced antioxidant defenses under drought, improving its stress tolerance (Zhang, 2010; Guan et al., 2022). Studies on *Solanum tuberosum* (Watkinson et al., 2006) and *Epimedium koreanum* (Wang, 2024) further support a strong correlation between flavonoid content and drought resistance. These findings align with the observed changes in flavonoid profiles in the roots of *I. stachyodes* seedlings under drought stress. Beyond direct ROS scavenging, flavonoids also contribute to drought adaptation by modulating phytohormone signaling, improving photosynthetic efficiency, and optimizing water uptake and utilization (Zhuang et al., 2023).

Based on the analysis of the flavonoid biosynthesis pathway, it was found that in the roots of *I. stachyodes* seedlings, the modulation of genes such as *CHS*, *C4H*, and *4CL* led to a significant down-regulation of most metabolites, including pinocembrin, gallocatechin, and catechin. Only the contents of garbunzol and dihydromyricetin showed a significant up-regulation. This selective accumulation phenomenon is consistent with the findings reported by in rice (Jayaraman et al., 2022). Previous studies have demonstrated that dihydromyricetin is a potent antioxidant capable of effectively scavenging ROS. Under drought conditions, plants may enhance their resistance to oxidative stress and improve drought tolerance by preferentially up-regulating such flavonoids with strong antioxidant activity (Huang et al., 2020). Furthermore, although flavanol metabolites such as gallocatechin and catechin exhibit strong antioxidant activity, they remained in a down-regulated state. This phenomenon may be associated with the suppression of this biosynthetic branch to conserve carbon sources and energy under extreme drought conditions. This finding aligns with the proposal that plants undergo metabolic reprogramming to cope with environmental stress under drought conditions (Kumar et al., 2021).

The isoflavonoid biosynthesis pathway displayed a similar accumulation pattern. Through regulation of key genes including *IFS*, *HIDH* and *I3’H*, the contents of major metabolites such as malonyldaidzin, formononetin 7-O-glucoside and daidzein-7-O-glucoside were significantly down-regulated, while only coumestrol, and medicarpin showed significant up-regulation. Studies have revealed that coumestrol enhances drought tolerance by activating the ABA signaling pathway to induce stomatal closure and reduce water loss (Tripathi et al., 2016). Medicarpin improves drought resistance through enhancing cell wall lignification to maintain water retention capacity under drought stress (Wang et al., 2022). As a crucial hydroxylated isoflavonoid derivative in leguminous plants, 2’-hydroxydaidzein is derived from daidzein hydroxylation. The introduction of a 2’-hydroxyl group significantly augments its free radical scavenging capacity, thereby enabling plants to effectively mitigate oxidative damage induced by drought stress (Fischer et al., 1990; Shao, 2020). These compounds participate in drought response by modulating cellular osmotic pressure or functioning as signaling molecules, exhibiting mechanisms analogous to *Gentiana rhodantha*’s response to drought stress (Ye et al., 2025). From this, it can be inferred that under drought stress, *I. stachyodes* adopts a regulatory strategy by prioritizing the synthesis of highly active defense compounds while suppressing energy-demanding secondary metabolic pathways, thereby redirecting carbon and nitrogen resources to maintain ribosomal function and enhance cell wall lignification. This metabolic reprogramming ensures the preservation of essential physiological functions under drought conditions. This regulatory mechanism shows similarities to the abiotic stress response strategies in woody plants (Yan et al., 2024) and wheat (Hu et al., 2018; Riaz et al., 2023).

Through combined transcriptomic and metabolomic analysis, it was found that drought stress primarily affects the flavonoid biosynthesis pathway. In response to the drought stress, some genes in the phenylpropanoid/flavonoid pathway such as *PAL*, *C4H*, *CCR*, and *DFR*, and their metabolites, i.e., dihydromyricetin, catechin, coumestrol, and formononetin 7-O-glucoside, experienced significant changes in expression and synthesis, respectively. This finding is consistent with previous studies on *Gossypium hirsutum* and *Ammopiptanthus mongolicus* under drought stress (Wu et al., 2014; Zhang, 2018). Through homology alignment analysis, these genes were found to have functionally conserved homologs in model plants such as *Arabidopsis thaliana*. For instance, *PAL* serves as the rate-limiting enzyme in the plant phenylpropanoid pathway and is extensively involved in flavonoid biosynthesis. Research has demonstrated that *PAL* exhibits clear homology with *AtPAL1/2*, with its function and regulatory mechanisms being highly conserved among plants (Dreßen et al., 2017; Chai et al., 2024). In this study, the key phenylpropanoid pathway enzyme *PAL* was significantly down-regulated to produce cinnamic acid, which serves as a crucial precursor for flavonoid biosynthesis. This regulatory pattern resembles that of the homologous genes *AtPAL1/2* in *A. thaliana*, demonstrating the highly conserved role of *PAL* in plant flavonoid biosynthesis (Huang et al., 2010).

Under drought stress, plants employ integrated physiological, biochemical, and molecular strategies to mitigate stress-induced damage and enhance survival. These adaptive responses can be broadly classified into: morphological modifications, metabolic reprogramming and molecular signaling regulation (Li, 2014). Among these, lignin deposition serves as a critical defense mechanism, bridging structural reinforcement and metabolic adaptation (Han et al., 2022). As a major component of the secondary cell wall, lignin enhances mechanical strength and confers resistance to abiotic stresses. Its biosynthesis involves two key reductases: *CCR* and *CAD*. Under drought conditions, coordinated up-regulation of *CCR* and *CAD* promotes lignin polymerization, leading to: enhanced cell wall lignification, reducing water loss through cuticular reinforcement, improved vascular bundle rigidity, maintaining hydraulic conductivity under water deficit, increased root mechanical strength, facilitating soil penetration and water uptake (Sun et al., 2022; Wu et al., 2022). Evidence from model and crop species in *A. thaliana*, drought-induced *CCR* expression and *CmCAD2/CmCAD3* up-regulation catalyze the conversion of cinnamaldehyde to cinnamyl alcohol, accelerating lignin monomer polymerization. This process preserves root structural integrity and xylem function under stress (Liu et al., 2020). *Populus davidiana* exhibits drought-responsive *CCR/CAD* up-regulation, reinforcing root cell walls to mitigate dehydration and maintain stability (Yang et al., 2023). In *Cucumis melo*, drought-triggered *CAD* overexpression enhances lignin biosynthesis, directly correlating with improved stress tolerance (Liu, 2019). In this study, significant *CCR* and *CAD* expression was detected in the phenylpropanoid biosynthesis pathway of *I. stachyodes*, suggesting a conserved lignin-mediated drought resistance mechanism.

## 5 Conclusion

In this study, metabolomics-transcriptomics technology was used to investigate the root response of *I. stachyoides* seedling under drought stress. It was found that under the condition of 0-5% relative field water, the number of detected genes and metabolites was the highest, and they were mainly enriched in the phenylpropanoid and flavonoid biosynthesis pathways. The phenylpropanoid and flavonoid biosynthesis pathways are the secondary metabolite synthesis pathways that actively respond to drought stress in the seedling roots. Metabolites such as p-coumaric acid, sinapine malate, eugenol, coumestrol, isosakuranetin, 2’-hydroxydaidzein, daidzein 7-O-glucoside-6″-O-malonate, formononetin 7-O-glucoside, 7-O-glucoside-6″-O-malonate, medicarpin, daidzein-7-O-glucoside, malonyldaidzin, pinocembrin, phloridzin chalcone, prunin, isosakuranetin, vitexin, gallocatechin, catechin, garbunzol and dihydromyricetin, as well as genes including *PAL*, *C4H*, *COMT*, *4CL*, *CHS*, *DFR*, *HIDH*, *I2’H*, *IF7GT*, *IF7MAT*, *IFR*, *VR*, *PTS* and *IFS*, are potential key substances for the roots of *I. stachyoides* seedlings to resist drought stress. Furthermore, as one of the effective components for the quality control of its medicinal materials, the content of catechins was significantly down-regulated under the condition of 0-5% relative field water holding capacity. Therefore, to improve the catechin content during the cultivation of *I. stachyoides*, attention should be paid to controlling soil moisture to avoid prolonged moderate or extreme drought conditions, as extreme drought can negatively affect the normal growth of *I. stachyoides*. At present, there are few studies on the molecular response mechanisms of drought treatment in *I. stachyoides* seedlings, and the available genetic information for reference is limited, which restricts the exploration of functional genes in *I. stachyoides* to some extent. In this study, the transcriptional and metabolic information of *I. stachyoides* roots under drought treatment was obtained, and the DEGs and DEMs in the flavonoid biosynthesis pathway were preliminarily investigated. This provides a reference for understanding the physiological responses of the seedlings to drought stress, and foundational data to facilitate the domestication of wild *I. stachyodes*.

## Data Availability

The original contributions presented in the study are included in the article/**Supplementary Material**. Further inquiries can be directed to the corresponding authors.
