# Supplementary material for: Transcriptomic and metabolomic analyses of root responses in Indigofera stachyodes seedlings under drought stress: a medicinal plant native to karst mountainous regions
Source: Front Plant Sci. 2025 Jul 1;16:1607789. doi: 10.3389/fpls.2025.1607789 (PMC12259631; doi:10.3389/fpls.2025.1607789)
Supplement: Supplementary file 1 [file Supplementaryfile1.zip › Supplementary Figure S3.DOCX]

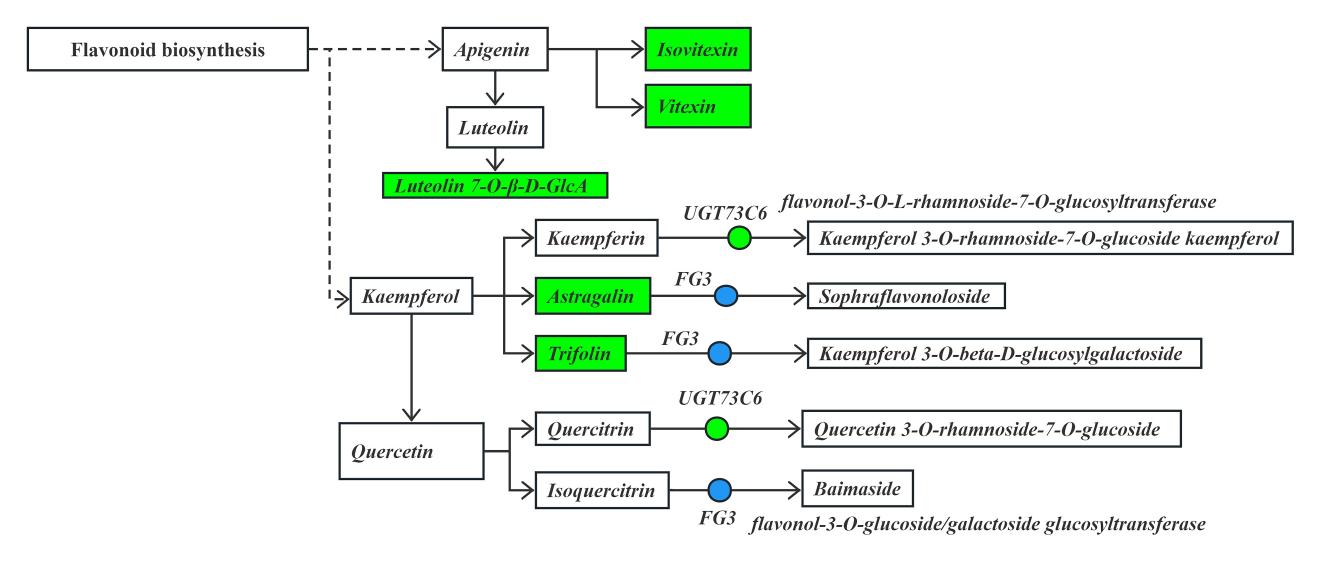


Supplementary Figure S3. The expression levels of DEGs and DEMs in the flavone and flavonol biosynthesis pathway. Note: The boxes represent DEMs, and the dots represent DEGs. Green indicates significantly downregulated, red indicates significantly upregulated, and blue indicates both up-regulation and down-regulation.
