# Supplementary material for: Transcriptomic and metabolomic analyses of root responses in Indigofera stachyodes seedlings under drought stress: a medicinal plant native to karst mountainous regions
Source: Front Plant Sci. 2025 Jul 1;16:1607789. doi: 10.3389/fpls.2025.1607789 (PMC12259631; doi:10.3389/fpls.2025.1607789)
Supplement: Supplementary file 1 [file Supplementaryfile1.zip › Supplementary Table S1.DOCX]

Supplementary Table S1. Statistics of transcriptome sequencing data

| 样品 | Clean Reads | Clean Base(G) | Q30(%) | GC Content(%) | Total mapped reads |
| --- | --- | --- | --- | --- | --- |
| ck-1 | 46 494 674 | 6.97 | 94.14 | 42.72 | 19 197 171(82.58%) |
| ck-2 | 40 834 030 | 6.13 | 94.03 | 43.32 | 17 362 425(85.04%) |
| ck-3 | 42 292 088 | 6.34 | 93.29 | 42.5 | 17 057 340(80.66%) |
| T0-1 | 67 350 608 | 10.1 | 93.95 | 44.3 | 29 371 807(87.22%) |
| T0-2 | 41 876 098 | 6.28 | 93.27 | 43.7 | 17 486 144(83.51%) |
| T0-3 | 45 848 184 | 6.88 | 93.94 | 43.86 | 19 736 731(86.10%) |
| T2-1 | 45 131 900 | 6.77 | 93.22 | 43.87 | 19 570 351(86.73%) |
| T2-2 | 45 217 790 | 6.78 | 92.91 | 42.44 | 19 293 935(85.34%) |
| T2-3 | 41 125 708 | 6.17 | 93.63 | 42.06 | 17 800 458(86.57%) |
