# Supplementary material for: Transcriptomic and metabolomic analyses of root responses in Indigofera stachyodes seedlings under drought stress: a medicinal plant native to karst mountainous regions
Source: Front Plant Sci. 2025 Jul 1;16:1607789. doi: 10.3389/fpls.2025.1607789 (PMC12259631; doi:10.3389/fpls.2025.1607789)
Supplement: Supplementary file 1 [file Supplementaryfile1.zip › Supplementary Table S2.DOCX]

Supplementary Table S2. Statistical table of DEGs

| Class Group | total | down | up |
| --- | --- | --- | --- |
| ck_vs_T0 | 7191 | 3422 | 3769 |
| ck_vs_T2 | 1264 | 766 | 498 |
| T2_vs_T0 | 3054 | 1486 | 1568 |
| total | 11 509 | 5674 | 5835 |
