# Supplementary figures and images for: Transcriptomic and metabolomic analyses of root responses in Indigofera stachyodes seedlings under drought stress: a medicinal plant native to karst mountainous regions
Source: Front Plant Sci. 2025 Jul 1;16:1607789. doi: 10.3389/fpls.2025.1607789 (PMC12259631; doi:10.3389/fpls.2025.1607789)

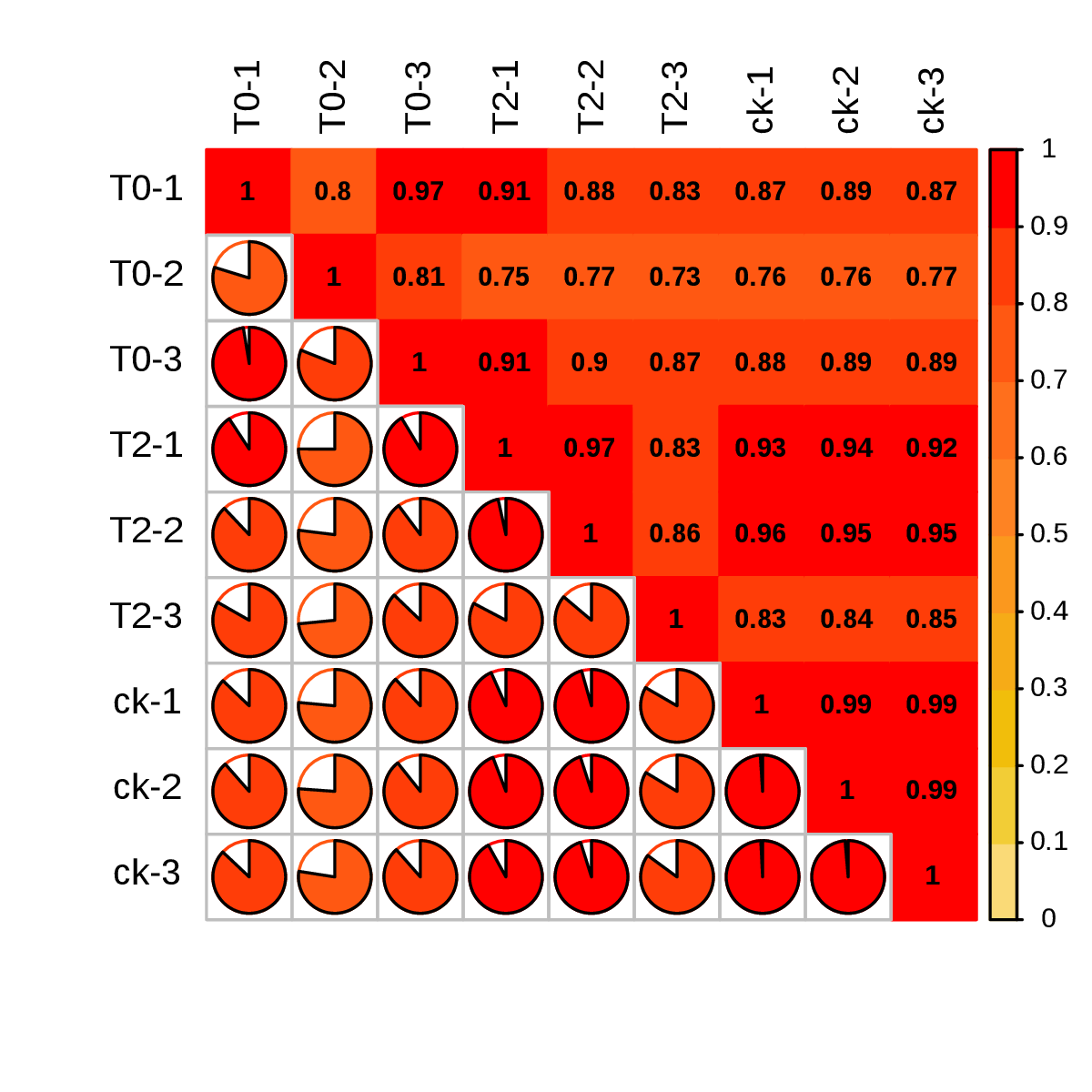


Supplementary Figure S1. Correlation analysis between samples

Supplement: Supplementary file 1 [file Supplementaryfile1.zip › Supplementary Figure S1.DOCX]

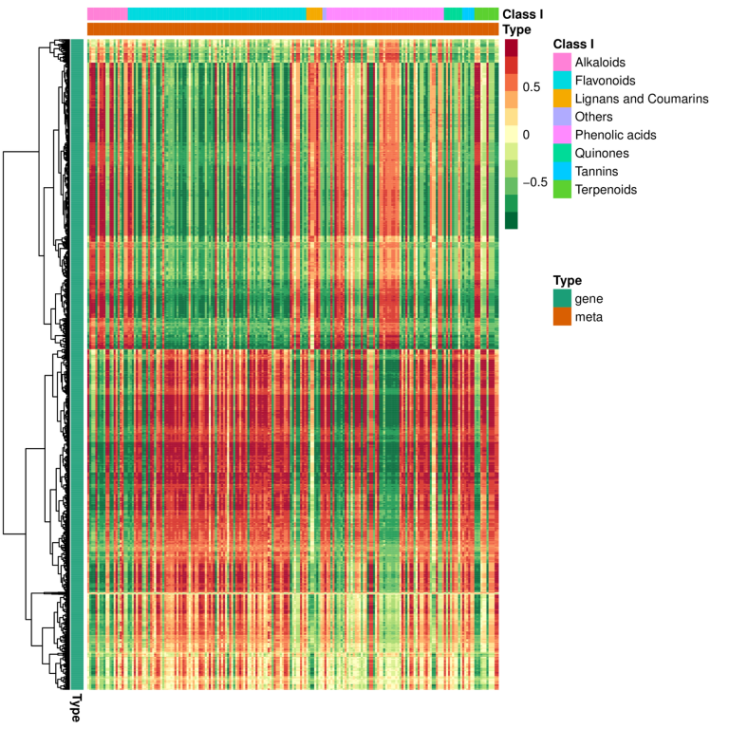


Supplementary Figure S2. Correlation analysis between DEGs and DEMs

Supplement: Supplementary file 1 [file Supplementaryfile1.zip › Supplementary Figure S2.DOCX]
